# DNA Repair Pathway in Ovarian Cancer Patients Treated with HIPEC

**DOI:** 10.3390/ijms24108868

**Published:** 2023-05-17

**Authors:** Dominika Flasarova, Katerina Urban, Ondrej Strouhal, Dusan Klos, Radmila Lemstrova, Pavel Dvorak, Pavel Soucek, Beatrice Mohelnikova-Duchonova

**Affiliations:** 1 Department of Oncology, Faculty of Medicine and Dentistry, Palacky University, 779 00 Olomouc, Czech Republic; 2 Department of Surgery I, Faculty of Medicine and Dentistry, University Hospital Olomouc, 779 00 Olomouc, Czech Republic; 3 Biomedical Center, Faculty of Medicine in Pilsen, Charles University, 323 00 Pilsen, Czech Republic; 4 Department of Toxicogenomics, National Institute of Public Health, 100 00 Prague, Czech Republic

**Keywords:** DNA repair, ovarian cancer, HIPEC, biomarkers

## Abstract

DNA repair pathways are essential for maintaining genome stability, and understanding the regulation of these mechanisms may help in the design of new strategies for treatments, the prevention of platinum-based chemoresistance, and the prolongation of overall patient survival not only with respect to ovarian cancer. The role of hyperthermic intraperitoneal chemotherapy (HIPEC) together with cytoreductive surgery (CRS) and adjuvant systemic chemotherapy is receiving more interest in ovarian cancer (OC) treatment because of the typical peritoneal spread of the disease. The aim of our study was to compare the expression level of 84 genes involved in the DNA repair pathway in tumors and the paired peritoneal metastasis tissue of patients treated with CRS/platinum-based HIPEC with respect to overall patient survival, presence of peritoneal carcinomatosis, treatment response, and alterations in the *BRCA1* and *BRCA2* genes. Tumors and metastatic tissue from 28 ovarian cancer patients collected during cytoreductive surgery before HIPEC with cisplatin were used for RNA isolation and subsequent cDNA synthesis. Quantitative real-time PCR followed. The most interesting findings of our study are undoubtedly the gene interactions among the genes *CCNH*, *XPA*, *SLK*, *RAD51C*, *XPA*, *NEIL1*, and *ATR* for primary tumor tissue and *ATM*, *ATR*, *BRCA2*, *CDK7*, *MSH2*, *MUTYH*, *POLB*, and *XRCC4* for metastases. Another interesting finding is the correlation between gene expression and overall survival (OS), where a low expression correlates with a worse OS.

## 1. Introduction

In 2020, according to the Global Cancer Observatory’s statistics, 313,959 women were newly diagnosed with ovarian cancer (OC) and nearly 185,000 died. The forecast for 2040 indicates that the incidence will increase to 434,184 and the mortality rate will increase to over 290,000 [1]. The danger of this disease is mainly in late diagnoses, with more than 70% of OC diagnosed at an advanced stage; therefore, this malignancy has the highest mortality rate of all gynecological cancers [2,3]. Additionally, due to the anatomical location of the ovaries, ovarian cancer is the most common neoplastic disease causing peritoneal metastases [4]. Currently, the gold standard for first-line chemotherapy is a treatment based on platinum derivatives (cisplatin/carboplatin) in combination with paclitaxel [5]. Neoadjuvant systemic chemotherapy is indicated for patients in whom primary surgical resection cannot be performed, optimally in three–four cycles, and systemic treatment is continued even after surgery [6]. Although OC is one of the most chemoresponsive tumors, the main obstacle to successful treatment and long overall survival is the development of drug resistance.

The presence of individually diverse multidrug resistance (MDR) to chemotherapy is the major challenge limiting the efficacy of systemic treatment and influencing its toxicity. The resistance of cells to anticancer drugs may be attributed to different mechanisms including abnormal membrane transport, ineffective metabolic drug conversion, enhanced metabolic inactivation, increased DNA repair, alteration of apoptotic pathways, enhanced desmoplastic reaction with paracrine activity, and activation of stem cells [7]. Intensive efforts have been made to clarify the mechanisms of platinum resistance in vitro [8], which can be divided into four groups: (i) mechanisms that reduce platinum accumulation; (ii) mechanisms that involve the intracellular inactivation of platinum compounds (e.g., coordination to thiol-containing biomolecules, such as glutathione and metallothioneins); (iii) mechanisms that enhance the repair of DNA damage; and (iv) mechanisms that block apoptosis. Thus, research addressing the pharmacogenomics of platinum compounds so far has focused mainly on interindividual differences in platinum pharmacokinetics and variations in genes coding transmembrane proteins, such as drug efflux ATP-binding cassette (ABC) and drug uptake solute carrier (SLC) transporters, and pathways involved in apoptosis (MAP kinase pathway and PI3K–AKT pathway) and DNA repair.

In 2018, the results of the first randomized controlled phase III trial were published to evaluate the effectiveness of cytoreductive surgery/hyperthermic intraperitoneal chemotherapy (CRS/HIPEC) in advanced OC. The OS of the patients was extended by almost 12 months due to the intraperitoneal administration of cisplatin warmed up to 42 °C during surgery [9].

HIPEC is used in conjunction with CRS for the removal of abdominal malignancies with peritoneal dissemination. Under normal conditions, the penetration of cytostatics into the abdominal cavity through the peritoneal membrane is difficult. The lavage of highly concentrated cytostatics directly to the tumor location significantly increases the penetration into the tumor tissue; in addition, hyperthermia already has a cytotoxic effect by itself. In such a way, the heating of cytostatics to 39–43 °C enhances their efficiency, activates heat shock proteins, and induces apoptosis and protein denaturation. Hyperthermia also affects the repair process of damaged DNA by accelerating the degradation of the BRCA2 protein. BRCA2 is essential for the repair of double-stranded DNA breaks by homologous recombination (HR), where it transfers RAD51 to the location of the damage. The RAD51 protein generates a break recovery process by copying information from an intact copy of damaged DNA [10,11,12]. By suppressing the DNA repair pathway, hyperthermia increases the effectiveness of treatment [13].

The purpose of this study was to provide better insight into the mechanism of resistance to platinum-containing HIPEC, and we evaluated changes in the expression of an 84-gene panel of DNA repair genes in primary tumor–peritoneal metastasis tissue pairs from ovarian cancer patients. We associated gene expression with *BRCA1/2* mutation status, overall survival, presence of peritoneal carcinomatosis, and treatment response.

## 2. Results

### 2.1. Study Population Characteristics

The study population consisted of 28 patients (the list of patients together with their characteristics is given in Table 1). The mean age of the patients was 57 years at the time of diagnosis with a range from 29 to 72 years. The majority of patients were high- grade serous carcinomas (HGSC) (78%) and had a mean peritoneal cancer index (PCI) of 12 (Table 1). The median progression-free survival (PFS) was 14 months, and the OS of patients after CRS/HIPEC was 30 months. The completeness of cytoreduction (CCR), which is an index quantifying the extent of residual disease after resection, ranged from 0 to 2. The majority of patients had CCR0 (>50%), while only one had CCR2.

### 2.2. Associations between Transcript Levels and BRCA1/2 Mutations

Mutations in *BRCA1/2* were present in 12 cases (46.2%), of which 8 patients had a mutation in *BRCA1* (66.7%) and 4 patients had a mutation in *BRCA2* (33.3%). From the heatmap (Figures S1 and S2), there were no significant expression profile changes between the wild-type and mutated samples.

### 2.3. Overall Survival and Progression-Free Survival

In our study, the median PFS of *BRCA1/2*-mutated patients was 12 months, while PFS patients without a *BRCA1/2* mutation had a median PFS of 14 months, but the trend does not reach statistical significance (*p* ≥ 0.05). The median OS of patients without a mutation was 29 months, while the median OS of patients with a *BRCA1/2* mutation was 32 months, but those results were not statistically significant either.

### 2.4. Correlation between Genes and Associations between Transcript Levels and Overall Survival

We observed a significant association between OS and the expression of six genes (*CCNH*, *MLH3*, *RAD51C*, *RPA3*, *SLK*, and *XPA*) in primary tumor tissues. The expression of these genes below the median predicted a significantly shorter OS (Figure S8). Additionally, *CCNH* expression correlates in a string with *XPA* and *SLK* and with *CDK7*, which correlates with *RAD51C* and *XPA*, correlating together in another string. All gene correlations were significant after a correction relative to multiple testing (padj ≤ 7.1 × 10^−6^). 

Due to greater variability in metastatic tissues, we also found a higher number of associations here. We observed a significant association between OS and the expression of 14 genes in the metastatic loci. The gene expression of most genes (*ATM*, *ATR*, *BRCA2*, *CDK7*, *MSH2*, *MUTYH*, *POLB*, and *XRCC4*) also showed the opposite relationship; i.e., the expression below the median is associated with shorter OS as in primary tumors (Figure S9). On the other hand, a high expression of six genes was associated with poor OS (*APEX1*, *PKRDC*, *RAD21*, *RAD23B*, *XRCC5*, and *XRCC6*) (Figure S9). The correlation analysis shows an interesting link between double-strand break repair (DSBR) and single-strand break repair (SSBR), including the mismatch repair (MMR) and base excision repair (BER) genes. *ATM–ATR–BRCA2* correlates with *CDK7*, *MSH2*, *MUTYH*, *POLB*, and *XRCC4* and forms very strong strings. Interactions among *MUTYH–MSH2, ATM–ATR*, and *ATM–BRCA2* are also supported by the STRING database, so they are the most interesting prognostic factors in our set. In contrast, the *APEX1*, *PKRDC*, *RAD21*, *RAD23B*, *XRCC5*, and *XRCC6* genes did not correlate with each other or with the above-mentioned DSB–SSB repair genes.

### 2.5. Associations between Transcript Levels and Peritoneal Carcinomatosis

The peritoneal cancer index is an important criterion for determining the extent of a tumor, its location, and the detection of distant metastases. Recurrence can be predicted based on the PCI value. According to Lampe et al. [14], in order to achieve CCR0, the cut-off PCI score for primary ovarian cancer should be <25. Patients with a *BRCA1/2* mutation had a median PCI of 7, while patients without the mutation had a higher median PCI of 13. On the heatmaps (Figures S3 and S4), there is a comparison of the expression of the individual genes of the DNA repair pathway stratified by the PCI of patients. In primary tumor tissue, more significant changes in expression are apparent compared to metastatic tissue. Nevertheless, no fundamental pattern indicating significant changes in expression levels, for example, in patients with high PCI, was observed.

### 2.6. Associations between Transcript Levels and Therapy

In terms of therapy, three parameters were considered—previous therapy, induction therapy, and the presence of residue after CRS. A total of 17 patients (60.7%) had not received previous treatment. Patients who had received previous treatment before CRS + HIPEC were in most cases treated with paclitaxel + carboplatin. Only seven patients (25%) did not receive induction treatments, and all other patients were treated with paclitaxel + CBDCA; only patients AF146 (Paclitaxel + CBDCA + Bevacizumab) and AF329 (Gemcitabine + CBDCA) had different induction treatments. According to the heatmap, there were no patterns to suggest that previous therapies, induction therapies, or CCR had a significant effect on expression changes in primary tumors (Figure S5) or metastatic tissues (Figure S6).

## 3. Discussion

This study aimed to provide biomarkers predicting platinum resistance. Our study offers an interesting insight into a broad area encompassing 84 genes of the DNA repair pathway and changes in this area in correlation with factors, such as overall survival, peritoneal carcinomatosis, therapy, or germline mutations in genes, that cause most hereditary ovarian cancers—*BRCA1* and *BRCA2*.

The most interesting finding in our study is the mutual correlation among the *ATM–ATR–BRCA2*, *CDK7*, *MSH2*, *MUTYH*, *POLB*, and *XRCC4* genes creating an interactive network and, additionally, its prognostic relevance for the specific set of patients under study. Moreover, some of these interactions are confirmed by the STRING database, specifically, *MUTYH*–*MSH2* (*MUTYH* represents the BER pathway, and *MSH2* represents the MMR pathway), *ATM*–*ATR* (*ATM* represents DSBR; *ATR* represents SSBR, and both genes ensure genome integrity), and *ATM*–*BRCA2* (these genes represent DSBR). Thus, this further substantiates their potential as prognostic biomarkers that would be worth further investigation in ovarian cancer. Some of the mentioned genes were already considered potential prognostic biomarkers for ovarian cancer in some earlier studies [15,16]. The OS of patients with a high expression of *ATM*, *ATR*, *BRCA2*, *CDK7*, *MSH2*, *MUTYH*, *POLB*, and *XRCC4* in metastases was significantly longer than in patients with low or no expression.

Guo et al. [17] reported that esophageal squamous cell carcinoma cells with downregulated *MUTYH* activity contribute to cisplatin resistance. Similarly to ovarian cancer, esophageal cancer also loses its initial sensitivity to platinum and develops resistance, suggesting that a similar mechanism to *MUTYH* could be at work in ovarian cancer. Resistance to platinum cytostatics results in poorer overall survival, and this correlates with our finding that low *MUTYH* expression corresponds to poorer OS.

In primary tumor tissue, the string correlations among *CCNH*, *XPA*, and *SLK* are interesting: *CCNH* and its correlation with *CDK7* and, last but not least, the correlation of *CDK7* in strings with *RAD51C* and *XPA*. *CCNH*, *CDK7*, and *XPA* represent the NER pathway and *RAD51C* represents the FA pathway. As with certain genes in metastatic tissue, the same phenomenon of interrelation between OS and expressions appears in tumor tissues. Specifically for the genes *CCNH*, *MLH3*, *RAD51C*, *RPA3*, *SLK*, and *XPA*, the low expression of these genes correlates with worse OS and vice versa. The same result for the *XPA* gene was also observed by Ganzinelli et al. [18].

When comparing tumor tissue vs. metastases (Figure S7), a preponderance of overexpression is evident. It seems that the first nine samples of metastatic tissue have the most pronounced expression changes; i.e., both the overexpression and underexpression of certain genes can be observed. The most pronounced overexpression is in the genes *CDK7*, *RPA3*, *ERCC5*, and *XPA* (nucleotide excision repair); *ATP23*, *ATR*, and *LIG4* (nonhomologous end joining); *NEIL1* (base excision repair); and *ATP23* and *ATR* (Supplementary data). However, overexpression is also evident in several other genes: *RPA1*, *XPC*, *RAD23A*, and *RAD23B* (nucleotide excision repair); *PRKDC*, *XRCC6*, and *XRCC5* (nonhomologous end joining); *TDG*, *APEX1*, and *PARP1* (base excision repair); *RAD50* and *MRE11A* (homologous recombination); *ATM* (genes defective in diseases associated with sensitivity to DNA-damaging agents); *MSH6* (mismatch repair); and *RAD21*, *SLK*, and *RFC1* (others). These genes form a cluster. A cluster is also shown for some underexpressed genes. Here, we can include base excision repair genes—*XRCC1*, *SMUG1*, *UNG*, *PARP2*, *PNKP*, and *PARP3*; homologous recombination genes—*DMC1*, *RAD54L*, *RAD51B*, *RAD52*, and *BRCA1*; nucleotide excision repair genes—*MMS19*, *LIG1*, *ERCC3*, *XAB2*, and *ERCC6*; *BRIP1* and *BRCA2*; *RAD18*—ubiquitination and modification; *POLD3*—DNA polymerases; and *TOP3B*—others. Brodsky in his publication states that metastatic and primary tumors have different expression profiles that reflect their state of proliferation and apoptosis, which also agrees with our study that the expression profile differs between tumor tissue and metastases. Thus, different pathways may be activated in metastases than in the primary tumors [19].

From the heatmap’s results, no significant association between the expression of DNA repair genes and *BRCA* mutation status was observed. However, more patients would be needed to draw final conclusions.

Tsibulak et al. [20] compared *BRCA1* and *BRCA2* mRNA expression in ovarian cancer tissues and noncancerous tissue in their study. An interesting feature of his study is that *BRCA1* expression was lower in the *BRCA1* mutants. This does not agree with our study in this respect, given that only two patients have a downregulation of the *BRCA1* genes in mutated *BRCA1* (AF185—AR, AF185—MR, and AF085—MR). However, the reason may be the small number of patients with a *BRCA1* mutation; thus, the result may be distorted. Similarly to our study, no significant expression changes in *BRCA1* and *BRCA2* were observed in the study by Olbromski et al. [21]. Osorio et al. [22] showed *OGG1* single nucleotide polymorphism association with the risk of ovarian cancer in BRCA1 carriers, but there was no correlation between *BRCA1* mutation and *OGG1* expression in our sample set.

In primary tumor tissue samples, the greatest changes in expression are mainly in AF050 and AF069 patients who have wild-type *BRCA1/2*. In these two samples, approximately half of the genes are overexpressed compared to other patients, with the smaller half showing significant underexpression. Apparent deregulation can also be observed in AF185, but there is already a mutation in *BRCA1*. The heatmap for metastatic tissue looks to be split in half with respect to overexpression and underexpression, perhaps with a slight preponderance of underexpression regardless of the *BRCA* mutation. From the heatmap’s results, no significant association between the expression of DNA repair genes and *BRCA* mutation status was observed. However, more patients would be needed to draw final conclusions. There are studies that report a better prognosis in patients with a *BRCA1/2* mutation due to a better response to platinum-derived chemotherapy; [23,24,25] in contrast, there are studies with the opposite opinion [26]. Some studies suggest that patient survival may be prolonged under specific conditions: For example, when the mutation is located in the *RAD51*-binding domain (exon 11) of the *BRCA2* gene [27]. The results of our study correspond to the first opinion that mutants have a better chance of survival. The median overall survival of patients without a *BRCA1/2* mutation was 29 months, while patients with a *BRCA1/2* mutation had an OS that was 3 months longer, thus resulting in 32 months (but it did not reach statistical significance). Alsop et al. reported that a mutation in *BRCA1/2*, in addition to improved overall survival compared to patients without the mutation, also had an effect on prolonging progression-free survival [28]. In our study, the median PFS of patients with a mutation in the *BRCA1/2* gene was 12 months, while patients without a mutation progressed after a median of 14 months.

According to the results of our study, we concluded that PCI and therapy seem to have no effect on the expression of DNA repair pathway genes.

Certain limitations of this study, such as the modest number of samples, need to be acknowledged, so our results require further confirmation in a larger cohort of patients. However, due to the fact that CRS together with HIPEC is a demanding surgical procedure and patient selection is strict, obtaining a sufficient number of patients is difficult. Despite the smaller number of patients, we reached interesting results justifying a more detailed study in the next phase. The benefit of our study is also the fact that it is the first of its kind to link the expression of DNA repair pathway genes with the results of HIPEC therapy in advanced ovarian cancer, so this constitutes hypothesis-generating research. In addition, this is a single-center study with homogeneously treated patients.

In the future, it would be beneficial to perform further verification analyses, especially at the level of protein expression and activity, and it would certainly be worthwhile to further study genes with significant mutual correlations.

## 4. Materials and Methods

### 4.1. Participants

Primary tumor and paired peritoneal metastatic tissue were removed from 28 patients with ovarian cancer who were treated with cytoreductive surgery and cisplatin-containing hyperthermic intraperitoneal chemotherapy between February 2018 and December 2019. Cisplatin was administered at 100 mg/m^2^ according to the platinum-sensitive patient protocol. The application time was 60 min. Immediately after cytoreduction, tissue samples were placed in RNA and later in a stabilization solution; they were then sent to a laboratory by a pneumatic tube where they were stored at −80 °C prior to processing. We also collected clinical data from medical patient records. All patients were asked to read and sign an informed consent form in accordance with the requirements of the institutional review boards of the Department of Oncology, Faculty of Medicine and Dentistry, Palacky University Olomouc and University Hospital Olomouc (protocol no. 118/17).

### 4.2. Isolation of RNA and cDNA Synthesis

Tissue samples were homogenized in liquid nitrogen. The total RNA was isolated using a Trizol reagent according to the manufacturer’s protocol (Invitrogen, Carlsbad, CA, USA). Aliquots were stored at −80 °C. The concentration of isolated RNA was measured using a UV BioSpectrometer (Eppendorf, Hamburg, Germany). The evaluation of RNA integrity was performed by denaturing electrophoresis. For the RNA sample to be continually used, two ribosomal subunits, 28S and 18S, had to be visible on the gel. Visualization was performed using the ChemiDoc MP Imaging system (Bio-Rad, Hercules, CA, USA). RNA samples passing quality checks were transcribed into complementary DNA (cDNA). The iScript gDNA Clear cDNA Synthesis Kit (Bio-Rad 172-5034), with nuclease-free water and 1 μg of RNA, was used for reverse transcription. The treatment of RNA samples with DNase I and subsequent cDNA synthesis were performed according to the manufacturer’s protocol (Bio-Rad, Hercules, CA, USA). The polymerase chain reaction (PCR) amplification of the ubiquitin C fragment was used to control the quality of the synthesized cDNA for possible genomic DNA (gDNA) contamination. The amplification product of ubiquitin C, cDNA, was 190 bp, while genomic DNA contained 1009 bp. Each sample was identified by the number and suffix -AR (tumor tissue) or -MR (metastatic tissue).

### 4.3. Quantitative Real-Time PCR

The level of expression of 84 DNA repair genes (Table S1) was measured by quantitative real-time PCR (qPCR). Five reference genes (ACTB, B2M, GAPDH, HPRT1, and RPLP0) were used for the normalization of results. To ensure consistent results, the entire quantitative analysis was performed in triplicate. PrimePCR SYBR Green Assays (Bio-Rad, Hercules, CA, USA) (Table S1) and SsoAdvanced Universal SYBR Green Supermix (Bio-Rad, Hercules, CA, USA) were used for PCR. The PCR reaction was run in 384-well plates, and the total volume of the reaction mixture per well was 10 µL. The concentration of cDNA in the reaction was 30 ng. The no primer control (NPC) contained nuclease-free water instead of the assay. The PCR reaction was started by initial denaturation at 95 °C for 30 s, followed by 40 cycles of denaturing for 5 s at 95 °C and elongation for 30 s at 60 °C. Following denaturation at 95 °C for 5 s, a melt curve was generated by heating from 65 °C to 95 °C with 0.5 °C increments, 5 s dwell time, and plate read.

### 4.4. Statistical Analyses

Creating principal component analysis (PCA) plots, hierarchical clustering and heatmap visualization were performed with the help of the publicly available bioinformatic web tool ClustVis [29]. Associations between categorized values, such as *BRCA1/2* mutation status and clinical data, were analyzed using the two-sided Fisher’s exact test. For the evaluation of continuous variables, such as gene expression against BRCA1/2 mutation status or clinical variables, the Kruskal–Wallis test was used. For the assessment of differences in gene expressions between both tumor loci types, we employed the paired Wilcoxon signed-rank test. Mutual gene expression correlations were analyzed by Spearman’s rho test. The correction for multiple testing was applied according to Benjamini and Hochberg [30]. Only correlations passing the multiple testing correction were further considered (padj ≤ 7.1 × 10^−6^). Survival functions were plotted by the Kaplan–Meier method and evaluated using the logrank test. The *p*-values were departures from two-sided tests. The above analyses were conducted in the statistical program SPSS v16.0 (SPSS, Chicago, IL, USA).

For the assessment of functional relations between correlating and clinically important genes, the STRING database of known and predicted protein–protein interactions with default settings was used [31].

### 4.5. Clinical Data

*BRCA* germline mutations were analyzed by the CZECANCA (CZEch CAncer paNel for Clinical Application; custom-made SeqCap EZ choice panel; Roche) panel, as previously described in detail [32].

Patient data concerning the peritoneal cancer index (PCI), which is an important criterion for determining the extent of a tumor, its location, and the detection of distant metastases, were correlated with the results. Clinicopathological data were obtained from patient records, namely, age, stage, grade, previous therapy, induction therapy, and presence of residue after CRS, as well as treatment outcomes, progression-free survival (PFS), and overall survival (summarized in Table 1). PFS was established as the time between the CRS and the proven recurrence or progression of the disease, and OS was established as the time between CRS and death. Furthermore, relationships between mutations and clinical data were monitored.

## Figures and Tables

**Table 1:** List of patients and their characteristics. CBDCA—carboplatin; HGSC—high-grade serous carcinoma; LGSC—low-grade serous carcinoma; CCA—clear cell adenocarcinoma; EC—endometroid carcinoma; GCT—granulosa cell tumor; SPA—serous papillary adenocarcinoma. †—dead.

ID	Tumor	Meta	Age	Histological Type	BRCA Status	Induction Therapy	CCR	PCI	Death	PFS (Months)	OS (Months)
AF050	AR	MR	29	HGSC	neg	Paclitaxel/CBDCA	1	30	alive	8	44
AF064	AR	MR	54	HGSC	neg	Paclitaxel/CBDCA	0	14	alive	18	38
AF069	AR	-	64	HGSC	neg	NO	0	14	alive	-	39
AF075	AR	-	61	HGSC	BRCA1	Paclitaxel/CBDCA	0	3	†	11	23
AF083	AR	MR	46	HGSC	BRCA2	NO	0	2	†	12	31
AF085	-	MR	35	HGSC	BRCA1	Paclitaxel/CBDCA	0	6	alive	18	39
AF096	AR	MR	48	HGSC	BRCA1	Paclitaxel/CBDCA	0	7	alive	26	32
AF102	AR	MR	56	HGSC	neg	NO	1	8	alive	17	39
AF111	AR	MR	60	HGSC	neg	Paclitaxel/CBDCA	0	6	alive	-	37
AF115	AR	-	55	HGSC	BRCA1	Paclitaxel/CBDCA	1	17	†	5	8
AF124	AR	-	51	HGSC	neg	Paclitaxel/CBDCA	0	8	alive	-	32
AF129	AR	MR	72	CCA	neg	NO	0	3	†	11	18
AF146	AR	MR	64	EC	neg	Paclitaxel/CBDCA/Bevacizumabe	1	11	alive	-	36
AF159	AR	MR	52	GCT	NA	NO	2	10	†	18	23
AF165	AR	MR	63	HGSC	BRCA1	Paclitaxel/CBDCA	1	18	†	10	13
AF174	AR	-	61	SPA	BRCA2	NO	0	3	alive	-	34
AF185	AR	MR	65	HGSC	BRCA1	Paclitaxel/CBDCA	1	12	alive	-	34
AF196	-	MR	64	HGSC	BRCA2	NO	0	3	alive	-	34
AF219	AR	MR	53	HGSC	neg	Paclitaxel/CBDCA	1	21	†	-	21
AF223	AR	MR	67	HGSC	neg	Paclitaxel/CBDCA	1	25	alive	7	23
AF230	AR	MR	62	HGSC	BRCA1	Paclitaxel/CBDCA	1	17	alive	7	32
AF231	AR	MR	67	HGSC	BRCA2	Paclitaxel/CBDCA	0	6	†	-	0
AF295	AR	MR	54	HGSC	BRCA1	Paclitaxel/CBDCA	0	15	alive	16	28
AF317	AR	MR	68	HGSC	neg	Paclitaxel/CBDCA	1	21	alive	22	25
AF329	AR	MR	50	HGSC	NA	Gemcitabine/CBDCA	1	18	alive	15	24
AF338	AR	-	59	HGSC	neg	Paclitaxel/CBDCA	0	0	alive	-	23
AF344	AR	MR	63	LGSC	neg	Paclitaxel/CBDCA	0	6	alive	-	22
AF365	-	MR	66	LGSC	neg	Paclitaxel/CBDCA	1	21	alive	-	21

## Data Availability

The data presented in this study are available upon request from the corresponding author.

## Supplementary Materials

The following supporting information can be downloaded at https://www.mdpi.com/article/10.3390/ijms24108868/s1.
