# Supplementary material for: DNA Repair Pathway in Ovarian Cancer Patients Treated with HIPEC
Source: Int J Mol Sci. 2023 May 17;24(10):8868. doi: 10.3390/ijms24108868 (PMC10219140; doi:10.3390/ijms24108868)
Supplement: Supplementary file 1 [file ijms-24-08868-s001.zip › ijms-2352551-supplementary.pdf]

Table S1. List of gene expression assay used for this study. BER - base excision repair; SDDA – genes defective in diseases associated with sensitivity to DNA damaging agents; HR – homologous recombination; FA – Fanconi anemia; NER - nucleotide excision repair; EPN – editing and processing nucleases; NHEJ – non-homologous end joining; DRD – direct reversal of damage; MMR - mismatch repair; DP - DNA polymerases; UM - ubiquitination and modification. Reference genes are marked with the symbol (*).

Gene ID	RefSeq	Assay ID	OMIM number	DNA repair pathways
APEX1	NM_080649	qHsaCID0012533	107748	BER
APEX2	NM_014481	qHsaCED0046611	300773	BER
ATM	NM_000051	qHsaCED0045448	607585	SDDA
ATP23	NM_033276	qHsaCID0008603	619760	Others
ATR	NM_001184	qHsaCID0022638	601215	Others
ATXN3	NM_004993	qHsaCED0037933	607047	Others
BRCA1	NM_007294	qHsaCED0038604	113705	HR
BRCA2	NM_000059	qHsaCED0044922	600185	FA
BRIP1	NM_032043	qHsaCED0044695	605882	FA
CCNH	NM_001239	qHsaCID0007234	601953	NER
CCNO	NM_021147	qHsaCED0002266	607752	Others
CDK7	NM_001799	qHsaCED0044530	601955	NER
DDB1	NM_001923	qHsaCID0021586	600045	NER
DDB2	NM_000107	qHsaCID0016961	600811	NER
DMC1	NM_007068	qHsaCID0017628	602721	HR
ERCC1	NM_001983	qHsaCID0008822	126380	NER
ERCC2	NM_000400	qHsaCID0037822	126340	NER
ERCC3	NM_000122	qHsaCED0046050	133510	NER
ERCC4	NM_005236	qHsaCID0018113	133520	NER
ERCC5	NM_000123	qHsaCED0044577	133530	NER
ERCC6	NM_000124	qHsaCED0048692	609413	NER
ERCC8	NM_000082	qHsaCED0042118	609412	NER
EXO1	NM_130398	qHsaCED0035328	606063	EPN
FEN1	NM_004111	qHsaCED0036761	600393	EPN
LIG1	NM_000234	qHsaCID0008449	126391	NER
LIG3	NM_002311	qHsaCID0018427	600940	BER
LIG4	NM_002312	qHsaCED0047042	601837	NHEJ
MGMT	NM_002412	qHsaCED0036780	156569	DRD
MLH1	NM_000249	qHsaCED0042382	120436	MMR
MLH3	NM_014381	qHsaCID0021156	604395	MMR
MMS19	NM_022362	qHsaCED0002239	614777	NER
MPG	NM_002434	qHsaCED0057200	156565	BER
MRE11A	NM_005590	qHsaCED0042016	600814	HR

MSH2	NM_000251	qHsaCED0042676	609309	MMR
MSH3	NM_002439	qHsaCED0043488	600887	MMR
MSH4	NM_002440	qHsaCED0042166	602105	MMR
MSH5	NM_002441	qHsaCED0038330	603382	MMR
MSH6	NM_000179	qHsaCED0043663	600678	MMR
MUTYH	NM_012222	qHsaCED0038608	604933	BER
NEIL1	NM_024608	qHsaCED0056614	608844	BER
NEIL2	NM_145043	qHsaCED0045633	608933	BER
NEIL3	NM_018248	qHsaCED0043285	608934	BER
NTHL1	NM_002528	qHsaCED0042216	602656	BER
OGG1	NM_002542	qHsaCED0043531	601982	BER
PARP1	NM_001618	qHsaCED0045162	173870	BER
PARP2	NM_005484	qHsaCED0044268	607725	BER
PARP3	NM_005485	qHsaCED0042084	607726	BER
PMS1	NM_000534	qHsaCID0022093	600258	MMR
PMS2	NM_000535	qHsaCED0043945	600259	MMR
PNKP	NM_007254	qHsaCID0014360	605610	BER
POLB	NM_002690	qHsaCED0056776	174760	DP
POLD3	NM_006591	qHsaCID0007030	611415	DP
POLL	NM_013274	qHsaCED0046360	606343	DP
PRKDC	NM_006904	qHsaCID0007587	600899	NHEJ
RAD18	NM_020165	qHsaCID0007443	605256	UM
RAD21	NM_006265	qHsaCED0046249	606462	Others
RAD23A	NM_005053	qHsaCED0037589	600061	NER
RAD23B	NM_002874	qHsaCED0005365	600062	NER
RAD50	NM_005732	qHsaCID0013265	604040	HR
RAD51	NM_002875	qHsaCED0042133	179617	HR
RAD51B	NM_133509	qHsaCID0036877	602948	HR
RAD51C	NM_058216	qHsaCED0036407	602774	FA
RAD51D	NM_002878	qHsaCED0047745	602954	HR
RAD52	NM_134424	qHsaCED0041872	600392	HR
RAD54L	NM_003579	qHsaCED0042535	603615	HR
RFC1	NM_002913	qHsaCED0042061	102579	Others
RPA1	NM_002945	qHsaCID0016650	179835	NER
RPA3	NM_002947	qHsaCED0023169	179837	NER
SLK	NM_014720	qHsaCED0042224	616563	Others
SMUG1	NM_014311	qHsaCED0037592	607753	BER
TDG	NM_003211	qHsaCED0004553	601423	BER
TOP3A	NM_004618	qHsaCED0047236	601243	SDDA
TOP3B	NM_003935	qHsaCID0008975	603582	Others
TREX1	NM_016381	qHsaCED0048594	606609	EPN
UNG	NM_003362	qHsaCED0042383	191525	BER
XAB2	NM_020196	qHsaCID0017153	610850	NER
XPA	NM_000380	qHsaCID0022359	611153	NER

XPC	NM_004628	qHsaCED0045651	613208	NER
XRCC1	NM_006297	qHsaCID0023107	194360	BER
XRCC2	NM_005431	qHsaCID0015694	600375	HR
XRCC3	NM_005432	qHsaCID0014128	600675	HR
XRCC4	NM_003401	qHsaCID0016093	194363	NHEJ
XRCC5	NM_021141	qHsaCID0011752	194364	NHEJ
XRCC6	NM_001469	qHsaCED0048082	152690	NHEJ
ACTB*	NM_001101	qHsaCED0036269	102630	
B2M*	NM_004048	qHsaCID0015347	109700	
GAPDH*	NM_002046	qHsaCED0038674	138400	
HPRT1*	NM_000194	qHsaCID0016375	308000	
RPLP0*	NM_001002	qHsaCED0038653	180510	

Figure S1. Heatmap of the association between transcript levels and *BRCA1/2* mutations in tumor tissue. BRCA 0 means wildtype, BRCA 1 means BRCA mutated, BER - base excision repair; DP - DNA polymerases; DRD – direct reversal of damage; EPN – editing and processing nucleases; FA – Fanconi anemia; HR – homologous recombination; MMR - mismatch repair; NER - nucleotide excision repair; NHEJ – non-homologous end joining; SDDA – genes defective in diseases associated with sensitivity to DNA damaging agents; UM - ubiquitination and modification.

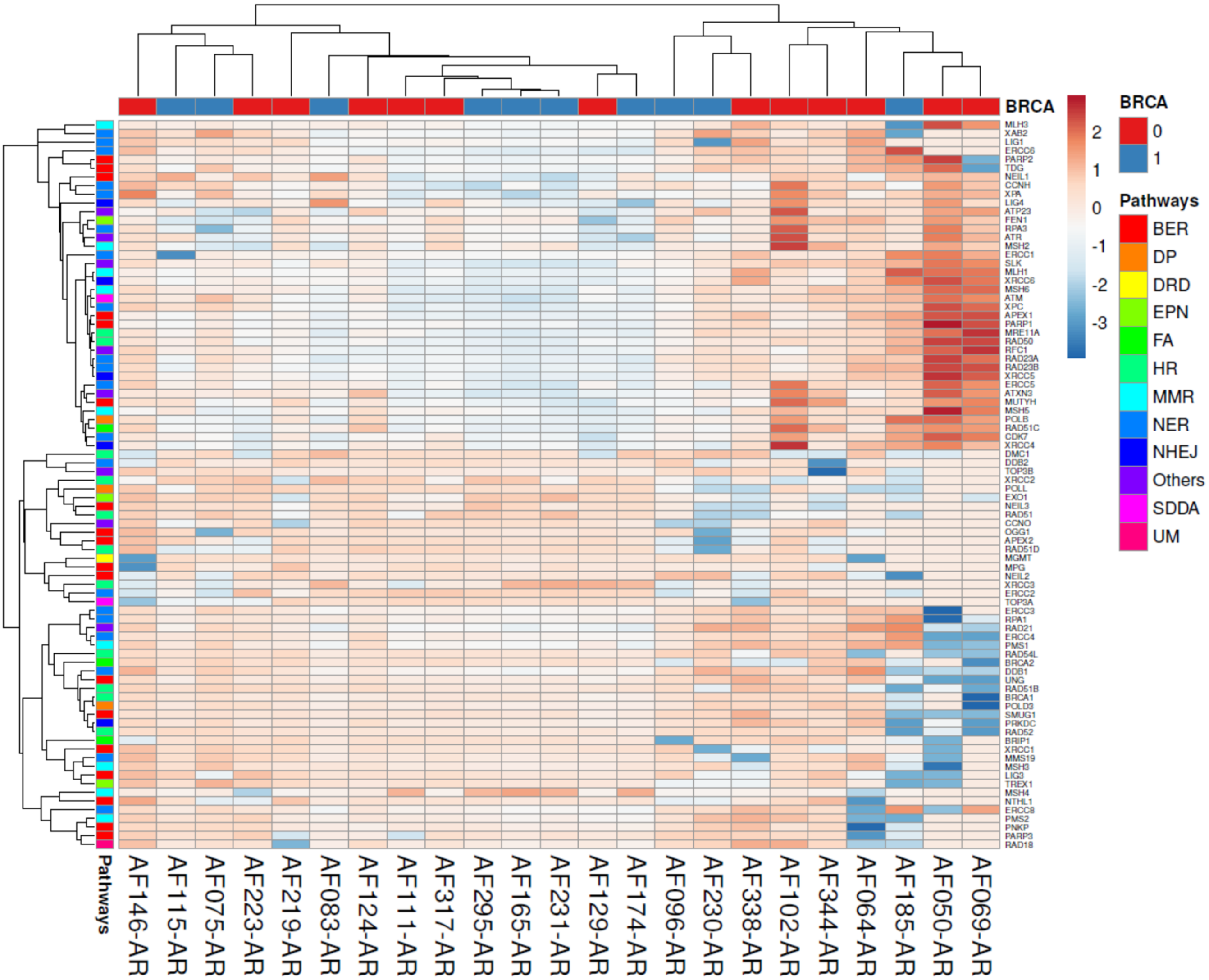

Figure S2. Heatmap of the association between transcript levels and *BRCA1/2* mutations in metastatic tissue.

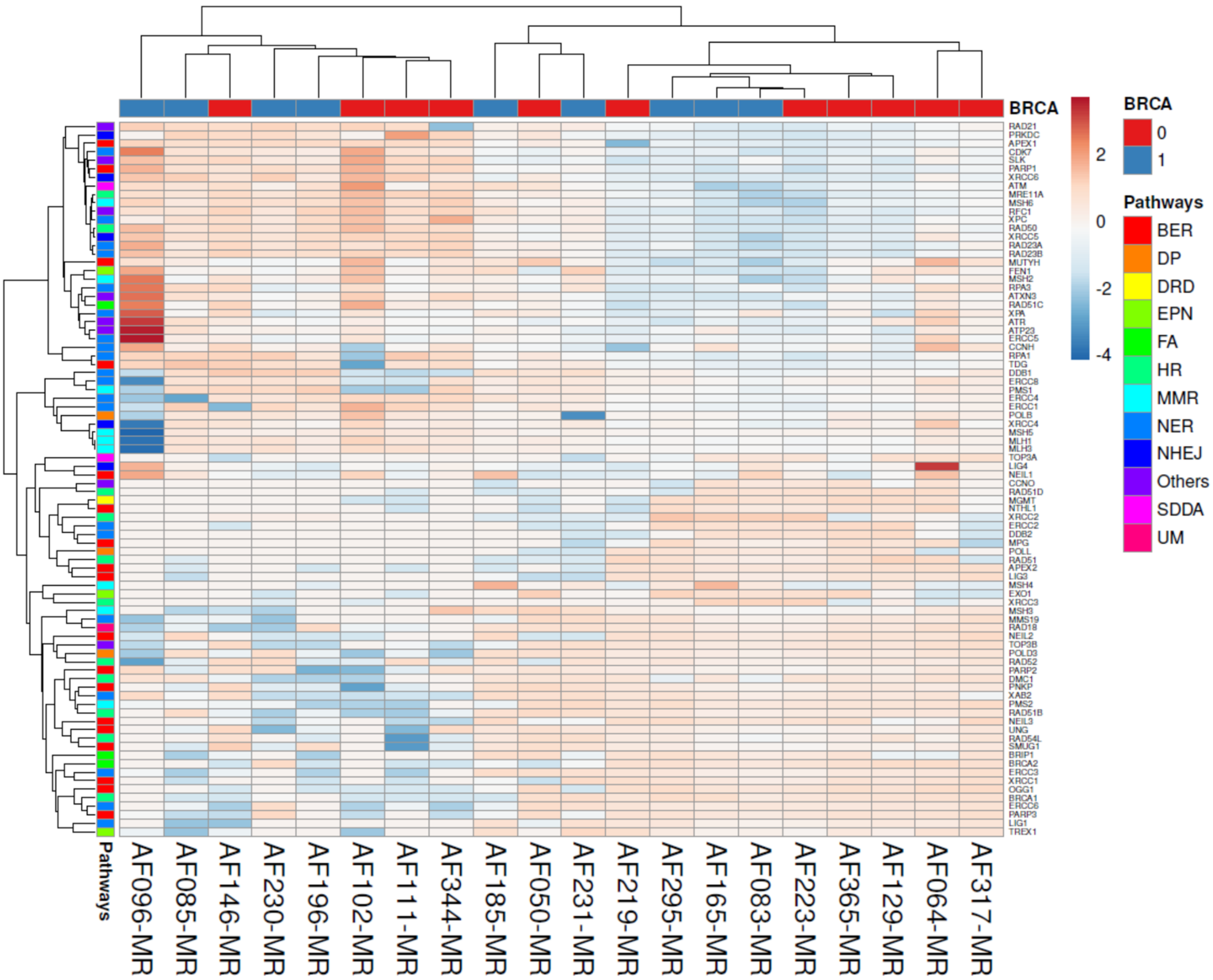

Figure S3. Heatmap of the association between transcript levels and peritoneal carcinomatosis in tumor tissue.

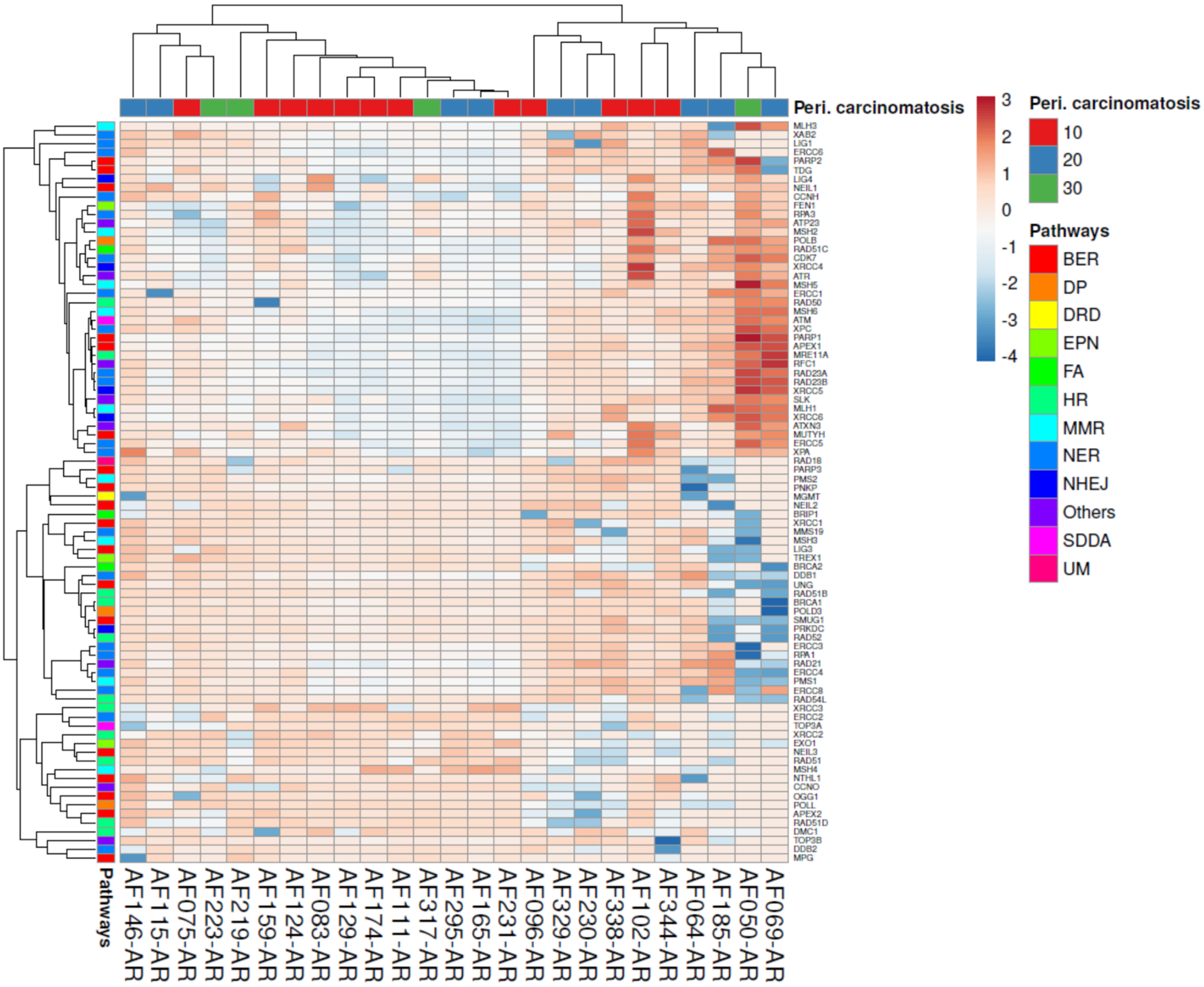

Figure S4. Heatmap of the association between transcript levels and peritoneal carcinomatosis in metastatic tissue.

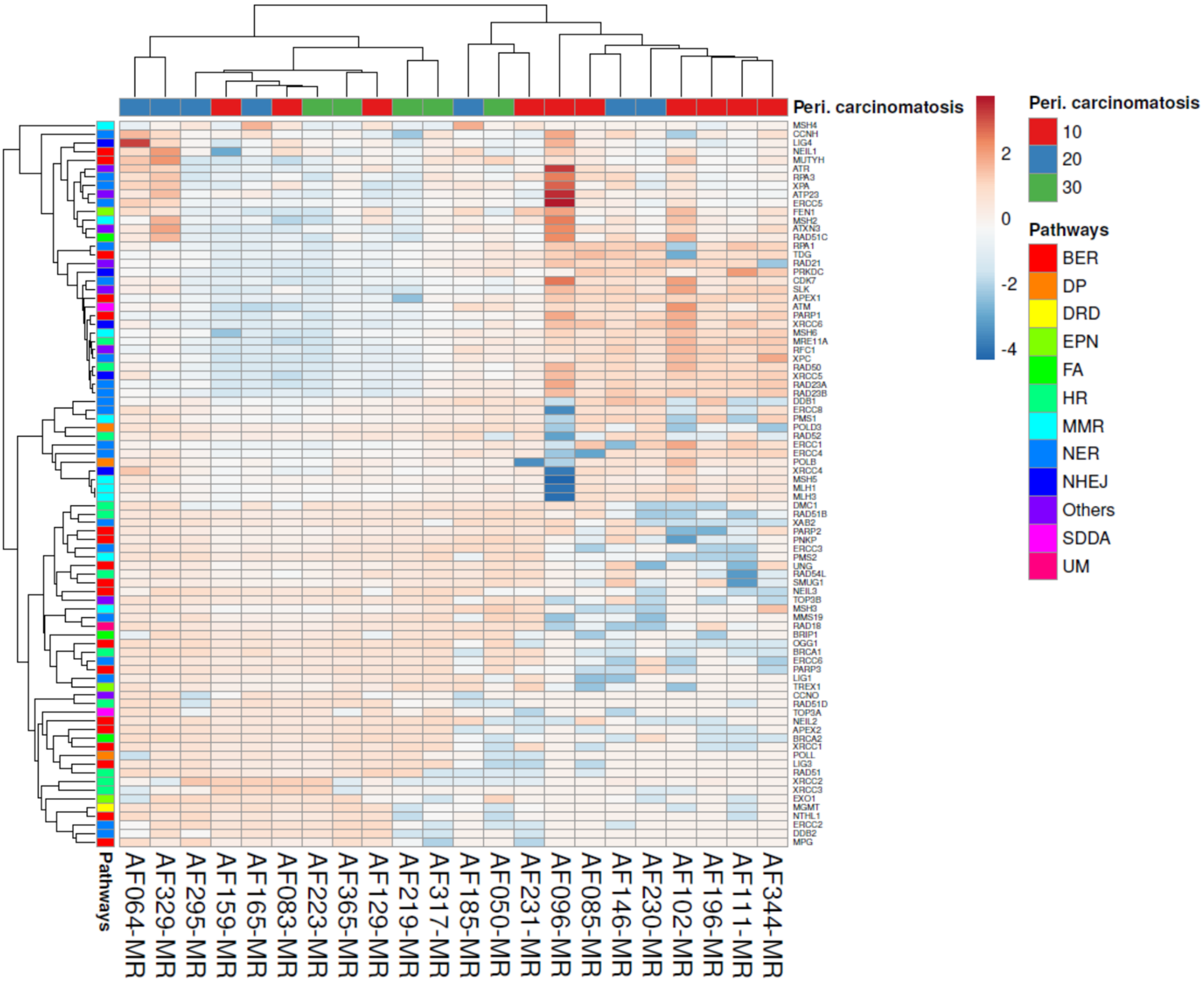

Figure S5. Heatmap of the association between transcript levels and therapy in tumor tissue.

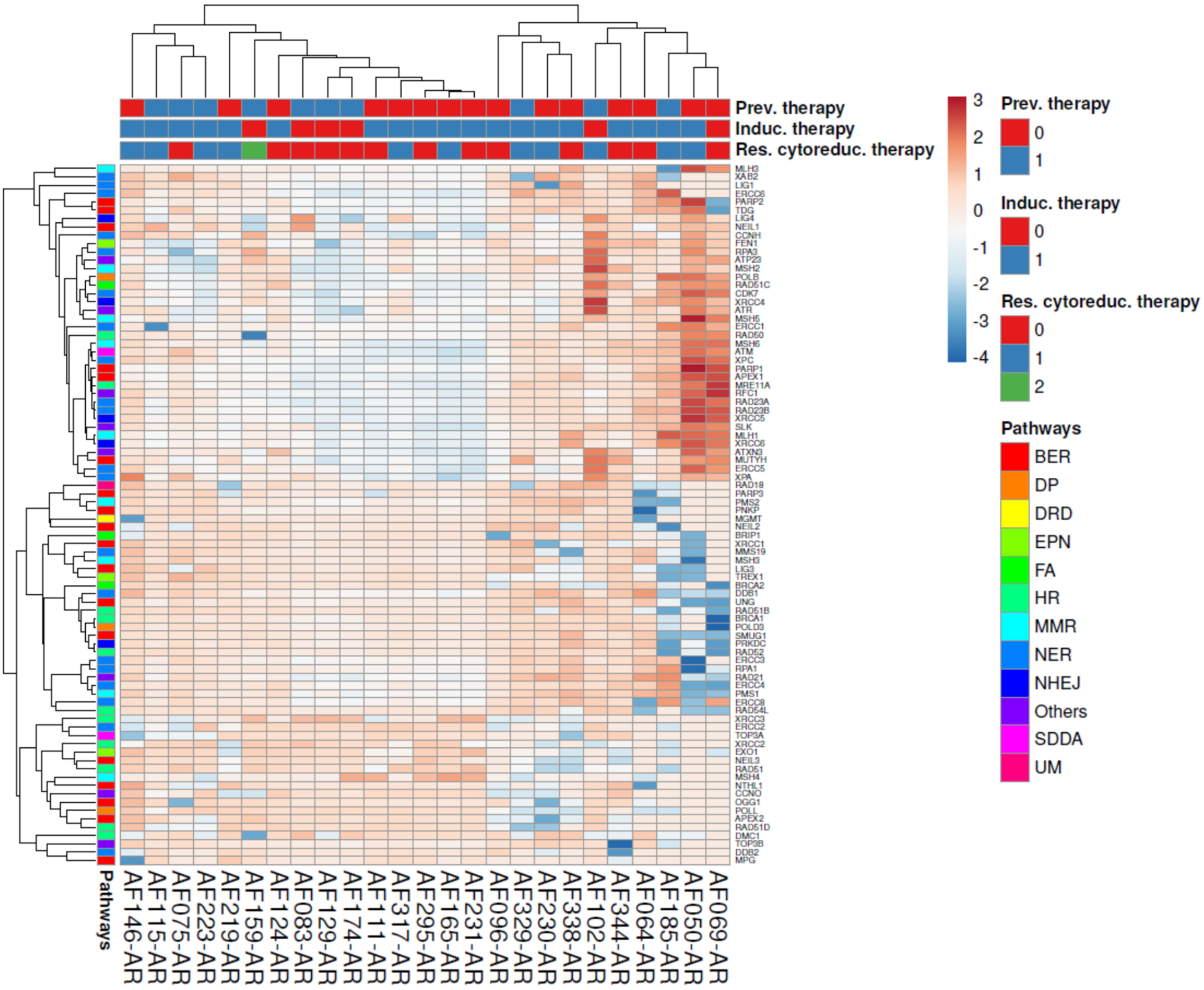

Figure S6. Heatmap of the association between transcript levels and therapy in metastatic tissue.

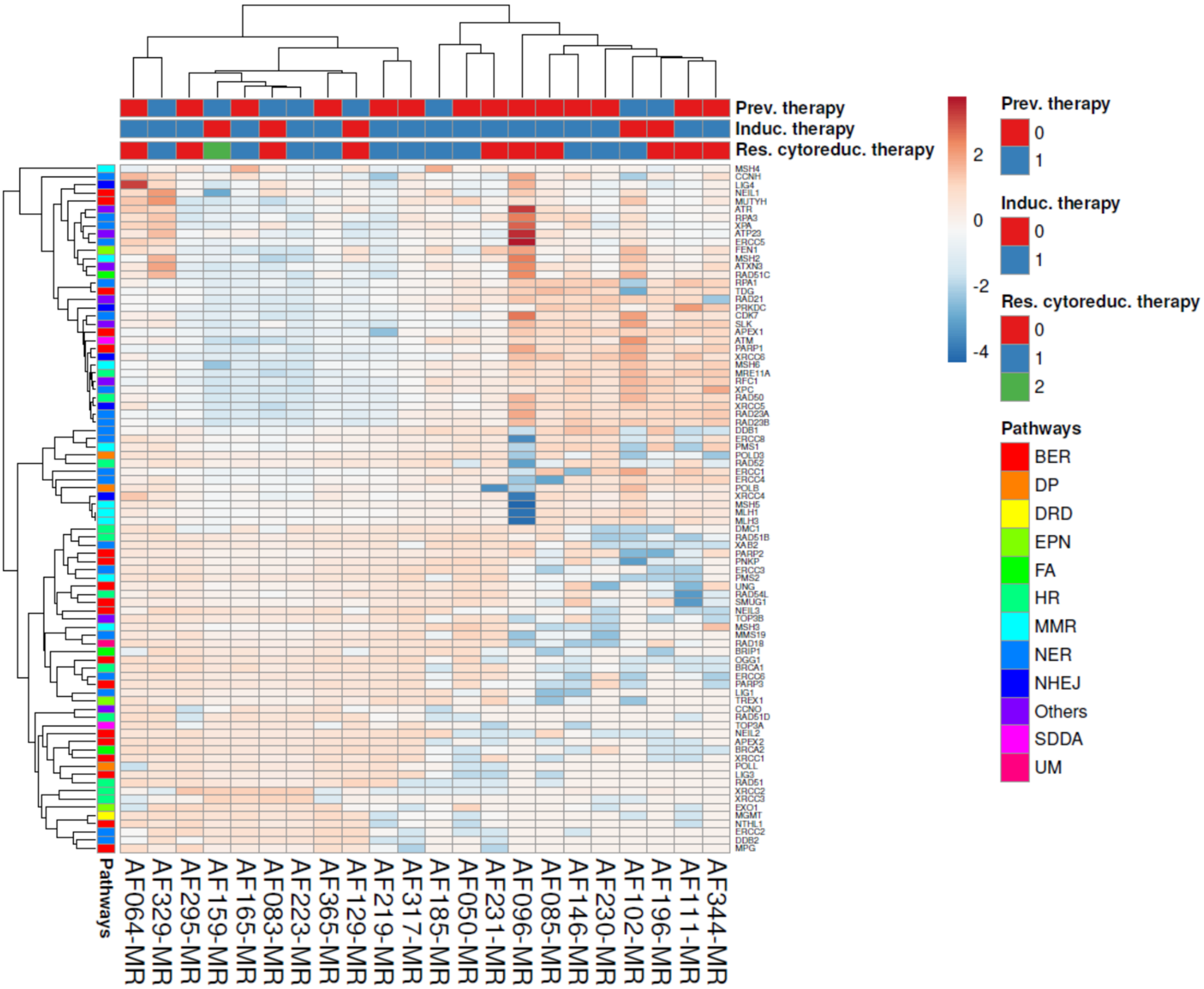

Figure S7. Heatmap comparing expression changes in tumor and metastatic tissue.

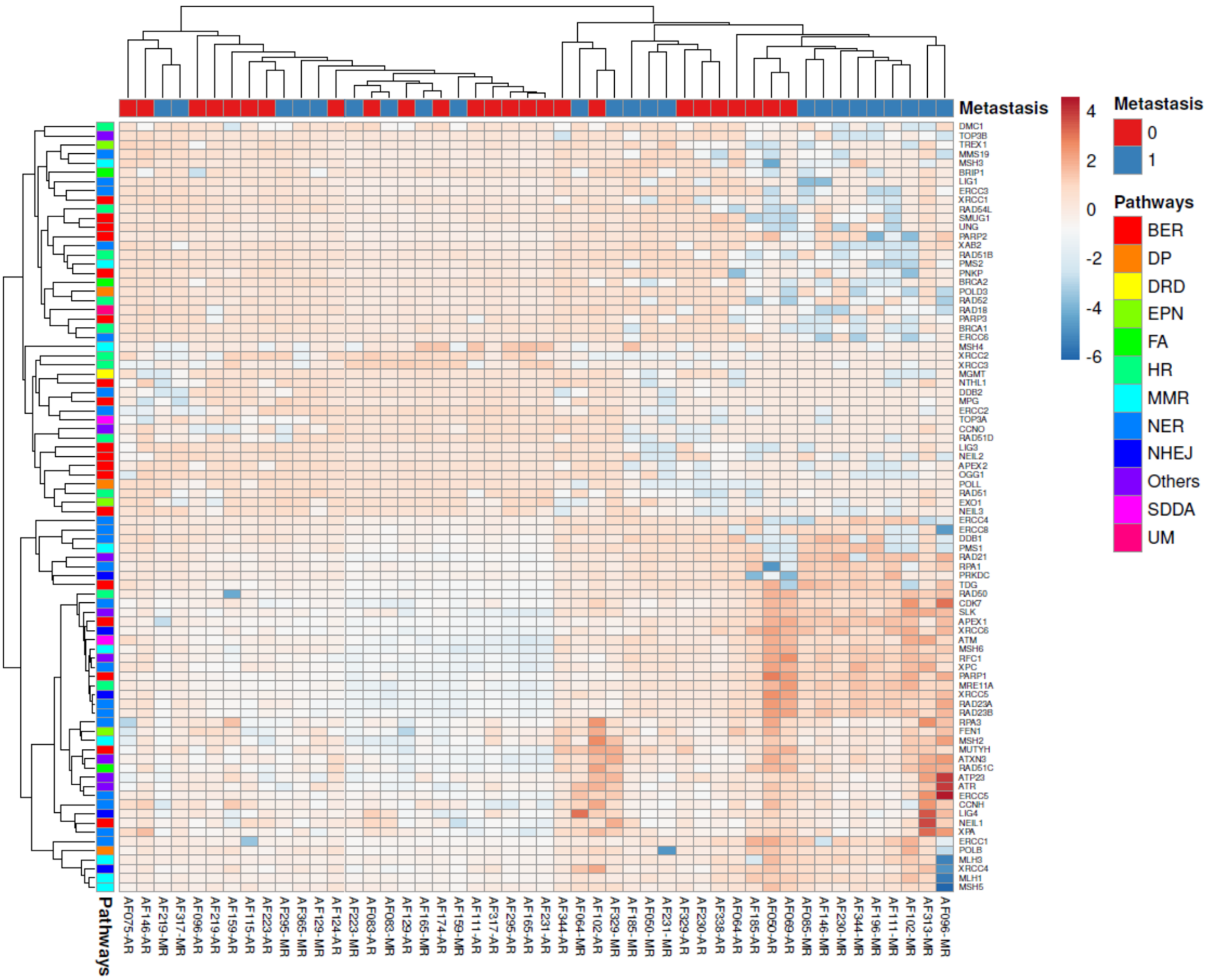

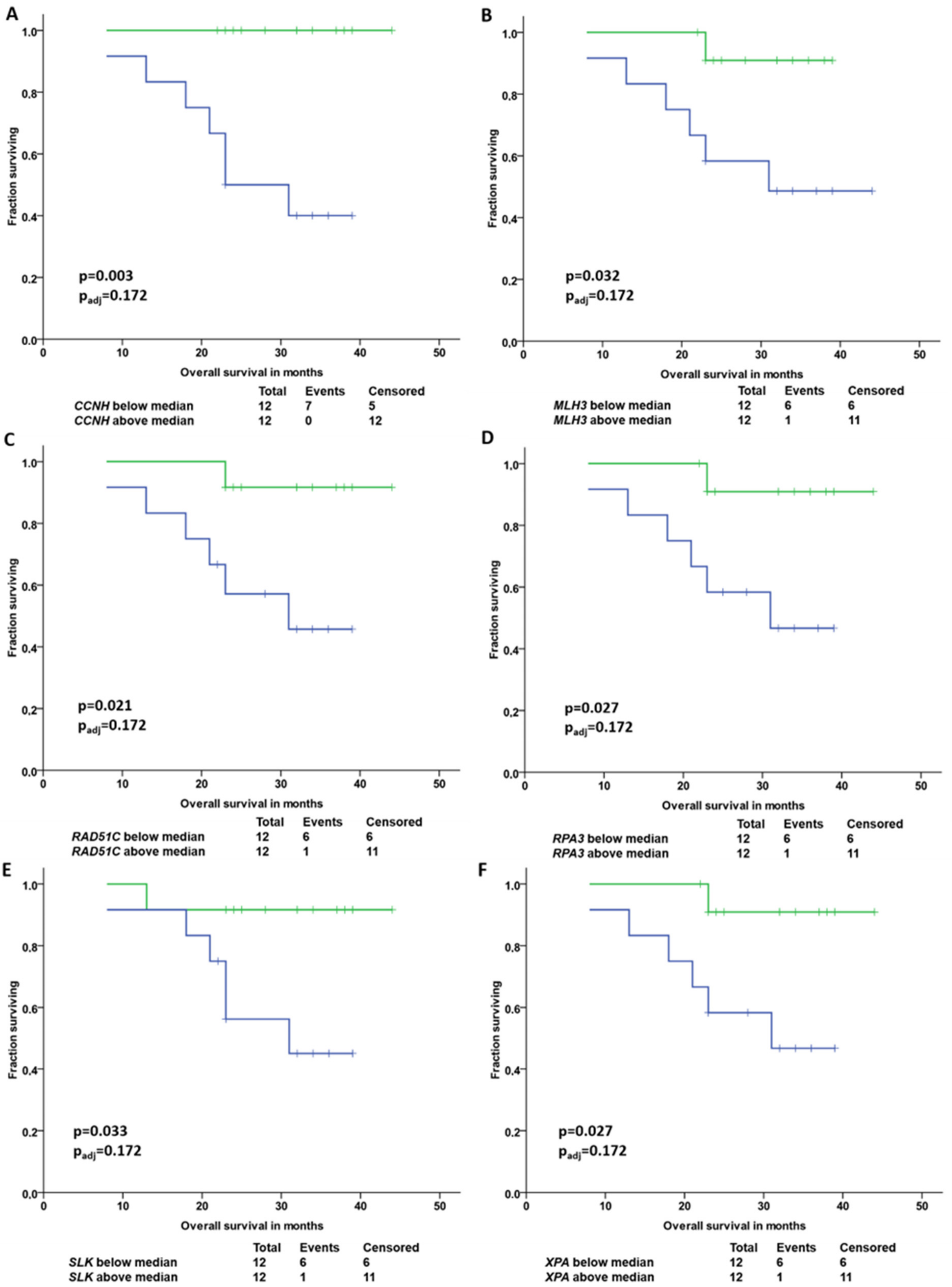

Figure S8. Significant associations of gene expression in primary tumor loci with overall survival Kaplan Meier survival curves for patient divided by median expression level of A - *CCNH*, B - *MLH3*, C - *RAD51C*, D - *RPA3*, E - *SLK*, and F - *XPA*. Green line - higher gene expression than median, blue line - lower gene expression than median.

A

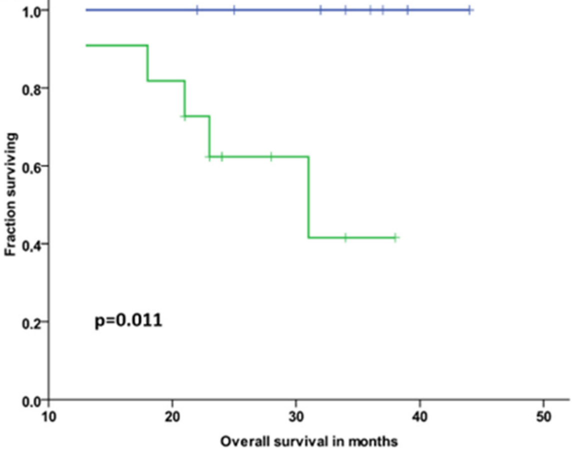

	Total	Events	Censored
APEX1 below median	10	0	10
APEX1 above median	11	5	6

B

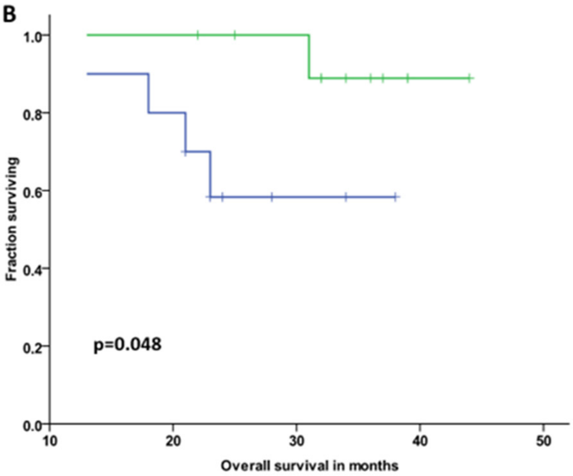

	Total	Events	Censored
ATM below median	10	4	6
ATM above median	11	1	10

C

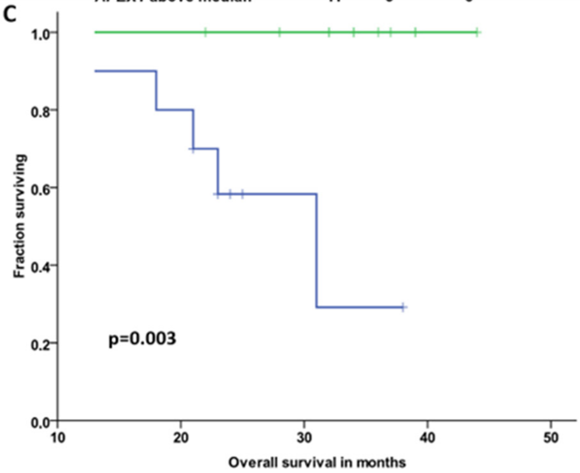

	Total	Events	Censored
ATR below median	10	5	5
ATR above median	11	0	11

D

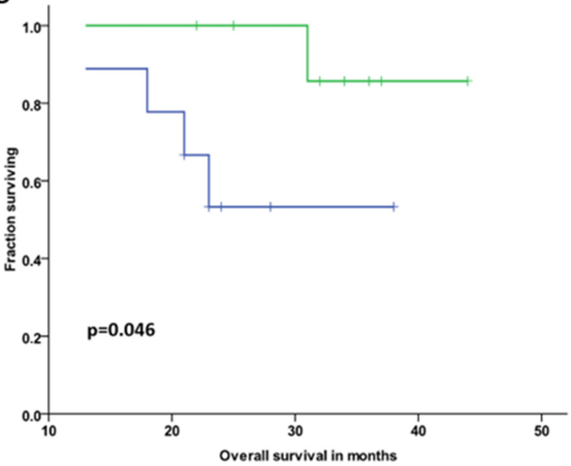

	Total	Events	Censored
BRCA2 below median	9	4	5
BRCA2 above median	9	1	8

E

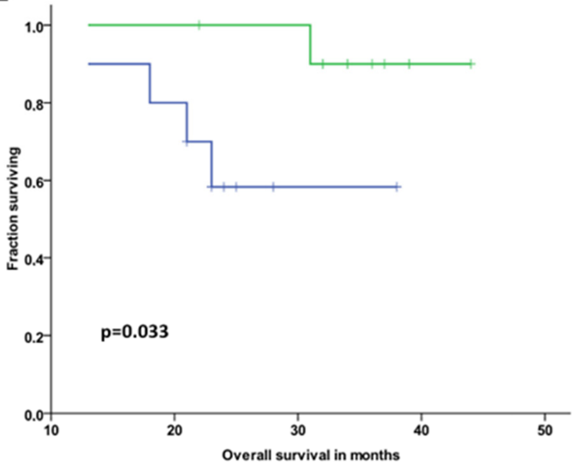

	Total	Events	Censored
CDK7 below median	10	4	6
CDK7 above median	11	1	10

F

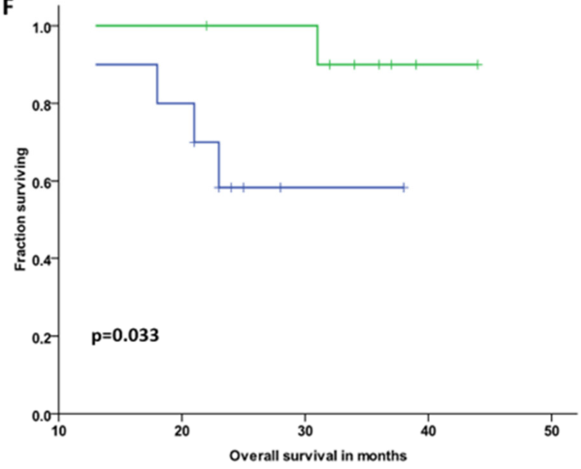

	Total	Events	Censored
MSH2 below median	10	4	6
MSH2 above median	11	1	10

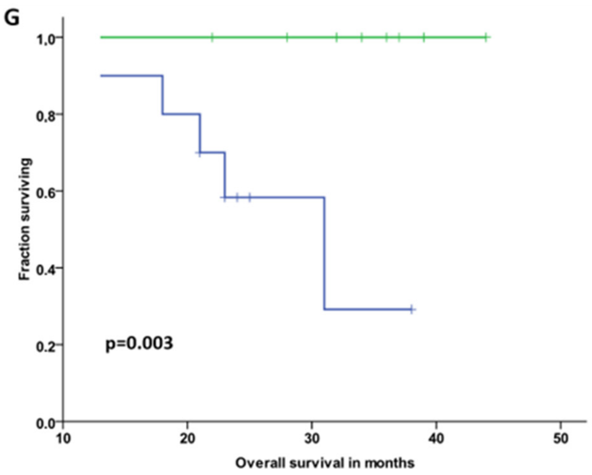

	Total	Events	Censored
***MUTYH* below median**	10	5	5
***MUTYH* above median**	11	0	11

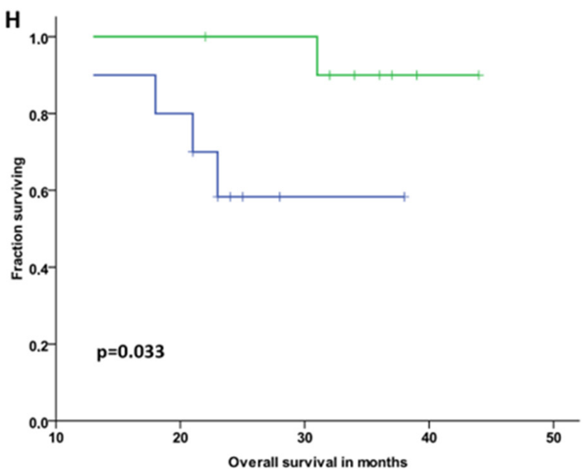

	Total	Events	Censored
***POLB* below median**	10	4	6
***POLB* above median**	11	1	10

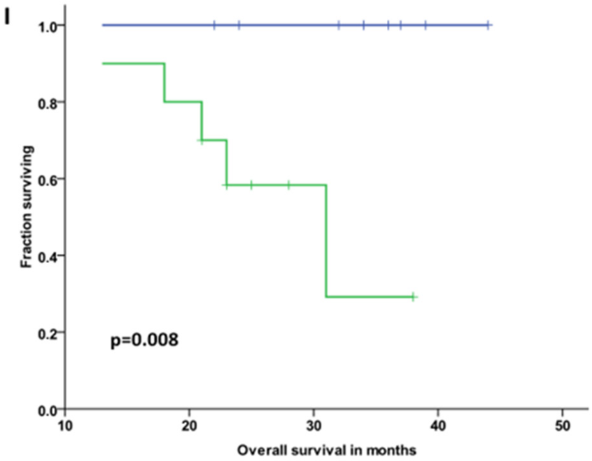

	Total	Events	Censored
***PRKDC* below median**	9	0	9
***PRKDC* above median**	10	5	5

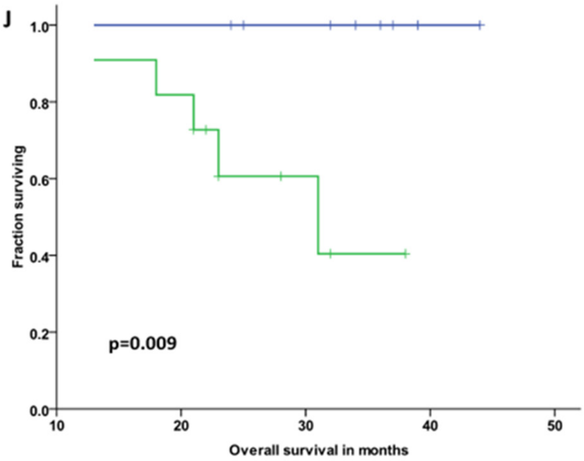

	Total	Events	Censored
***RAD21* below median**	10	0	10
***RAD21* above median**	11	5	6

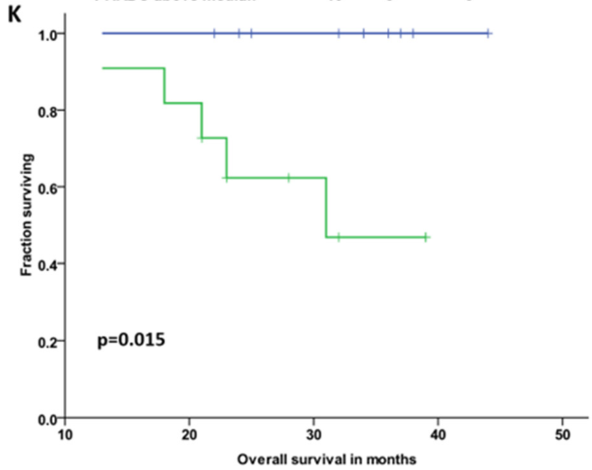

	Total	Events	Censored
***RAD23B* below median**	10	0	10
***RAD23B* above median**	11	5	6

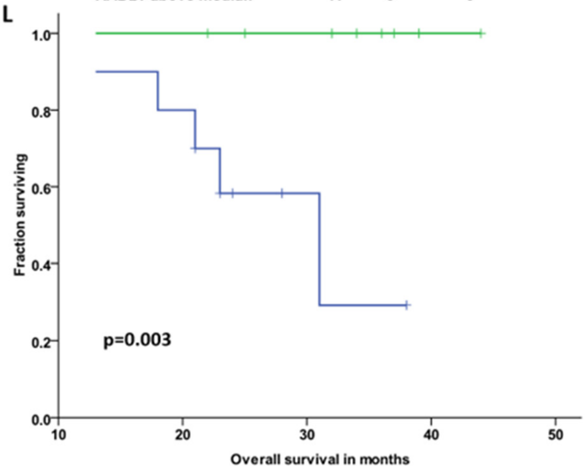

	Total	Events	Censored
***XRCC4* below median**	10	5	5
***XRCC4* above median**	11	0	11

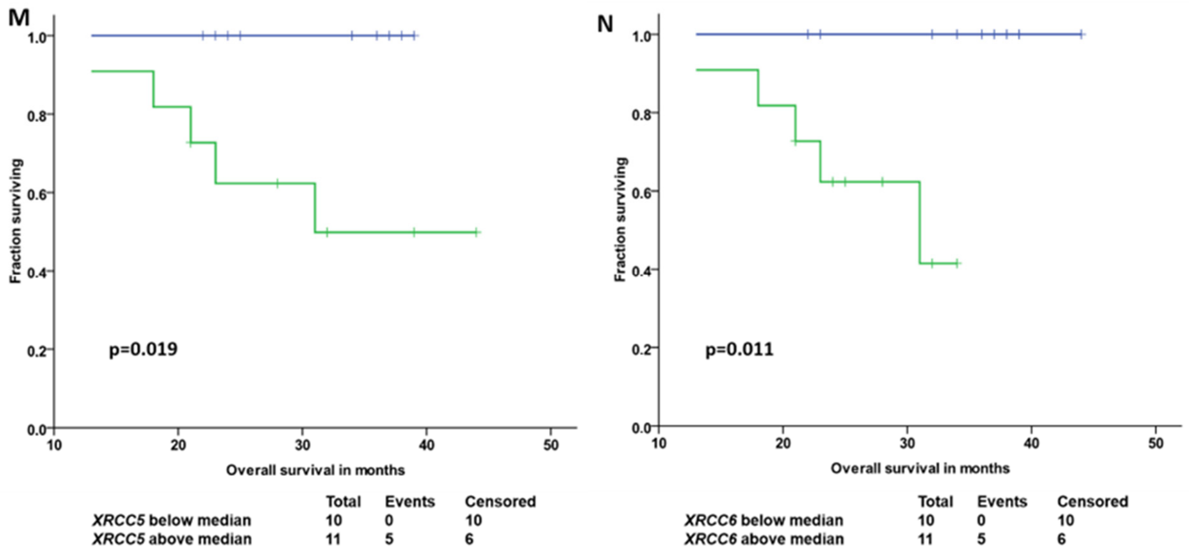

Figure S9. Significant associations of gene expression in metastatic loci with overall survival Kaplan Meier survival curves for patients divided by median expression level of A – *APEX1*, B - *ATM*, C - *ATR*, D – *BRCA2*, E – *CDK7*, F – *MSH2*, G – *MUTYH*, H - *POLB*, I - *PRKDC*, J – *RAD21*, K – *RAD23B*, L – *XRCC4*, M – *XRCC5*, and N – *XRCC6*. Green line - higher gene expression than median, blue line - lower gene expression than median.
